# Partial Inhibition of RNA Polymerase I Promotes Animal Health and Longevity

**DOI:** 10.1016/j.celrep.2020.01.017

**Published:** 2020-02-11

**Authors:** Guillermo Martínez Corrales, Danny Filer, Katharina C. Wenz, Abbie Rogan, George Phillips, Mengjia Li, Yodit Feseha, Susan J. Broughton, Nazif Alic

**Affiliations:** 1 Institute of Healthy Ageing and the Research Department of Genetics, Evolution, and Environment, University College London, WC1E 6BT London, UK; 2 Division of Biomedical and Life Sciences, Faculty of Health and Medicine, Lancaster University, LA1 4YQ Lancaster, UK

**Keywords:** RNA polymerase I, aging, ribosomal RNA/DNA, *Drosophila*, old-age health, lifespan, longevity

## Abstract

Health and survival in old age can be improved by changes in gene expression. RNA polymerase (Pol) I is the essential, conserved enzyme whose task is to generate the pre-ribosomal RNA (rRNA). We find that reducing the levels of Pol I activity is sufficient to extend lifespan in the fruit fly. This effect can be recapitulated by partial, adult-restricted inhibition, with both enterocytes and stem cells of the adult midgut emerging as important cell types. In stem cells, Pol I appears to act in the same longevity pathway as Pol III, implicating rRNA synthesis in these cells as the key lifespan determinant. Importantly, reduction in Pol I activity delays broad, age-related impairment and pathology, improving the function of diverse organ systems. Hence, our study shows that Pol I activity in the adult drives systemic, age-related decline in animal health and anticipates mortality.

## Highlights

• Partial inhibition of RNA polymerase I (Pol I) can extend lifespan in the fruit fly
• Reducing Pol I activity after development and only in the gut is sufficient
• Pol I activity affects aging from both post-mitotic and mitotically active cells
• Pol I activity affects the age-related decline in performance of multiple organs

## Graphical Abstract

**Figure:**
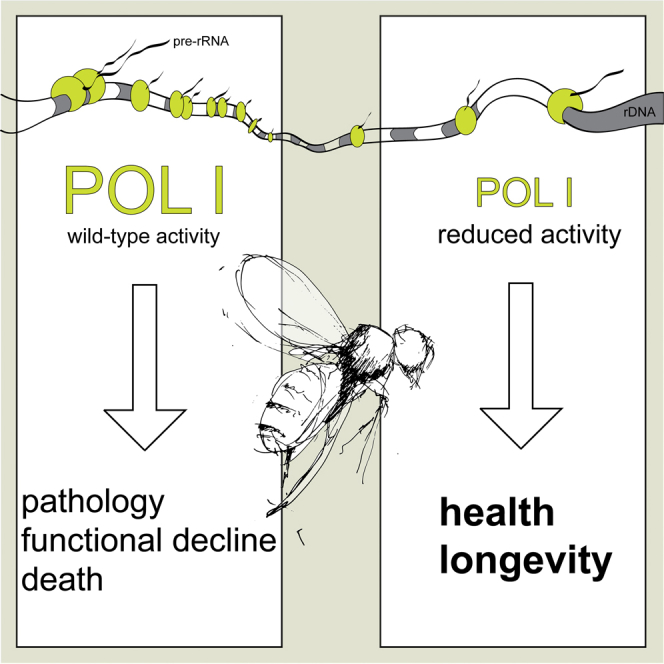

## Teaser

RNA polymerase I is a conserved eukaryotic enzyme that transcribes a single gene to generate precursor ribosomal RNA. Martínez Corrales et al. show that reducing the activity of this polymerase, even only in subsets of adult cells, can promote broad health benefits and longevity in the animal model *Drosophila melanogaster*.

## Introduction

Most animals age; their vitality, health, and survival decline as they get older (Martínez, 1998). In humans, age is the main risk factor for the predominant killer and debilitating diseases, including cancer, cardiovascular disease, and neurodegeneration (Christensen et al., 2009, Fontana et al., 2010, López-Otín et al., 2013, Niccoli and Partridge, 2012). Because the proportion of older people in many populations is increasing at an alarming rate (Christensen et al., 2009), identifying mechanisms that can be harnessed to improve human health and well-being in old age is an urgent priority for biomedical research. Over the last three decades, research in basic biogerontology has shown that aging is moldable by identifying fundamental cellular and organismal processes that can be manipulated to extend a healthy lifespan (Alic and Partridge, 2011, López-Otín et al., 2013, Partridge et al., 2018). These processes can often be altered in the adult to achieve longevity by reprogramming gene expression via transcriptional regulation.

The task of transcription in the eukaryotic nucleus is divided between three nuclear RNA polymerases (Pols) (Roeder and Rutter, 1969). Pol I, Pol II, and Pol III have distinct subunit composition, have distinct biochemical properties, and transcribe distinct classes of genes (Roeder and Rutter, 1969, Werner and Grohmann, 2011, Vannini and Cramer, 2012). Pol II-generated transcripts include all protein-coding messenger RNAs (mRNAs) and perform a vast number of cellular functions. The historical focus on Pol II is also observed in aging research, in which the most effort has been invested in understanding how a number of Pol II transcription factors guide pro-longevity transcriptional programs (e.g., Murphy et al., 2003, Hsu et al., 2003, Tepper et al., 2013, Alic et al., 2011, Alic et al., 2014, Dobson et al., 2019). The other two Pols have received much less attention, despite their fundamental cellular roles. We have recently described an evolutionarily conserved role for Pol III in aging (Filer et al., 2017). However, the role of Pol I remains unexplored.

Pol I is the fundamental structurally and functionally conserved eukaryotic enzyme that transcribes a single gene, ribosomal DNA (rDNA) (Vannini and Cramer, 2012). It generates the pre-rRNA that is processed into the mature 18S, 5.8S, and 28S rRNAs, the key structural and catalytic components of the ribosome (Grummt, 2003, Kressler et al., 2017). Even though Pol I has only one task to perform, its activity accounts for a major portion of cellular transcription due to the high cellular demand for rRNA (Grummt, 2003). rDNA is present in hundreds of copies per genome that are organized in large arrays of tandem repeats in one or several genomic locations (Grummt, 2003). Pol I is known to be essential for cellular growth and proliferation and for organismal growth, and it is often deregulated in cancers (Drygin et al., 2010, Grewal et al., 2007, Ghosh et al., 2014, Sriskanthadevan-Pirahas et al., 2018). The focus on this growth-promoting role of Pol I has often precluded investigating its potential function(s) in more complex cellular and animal traits. However, recent work has generated unsuspected insights. For example, Pol I activity in one tissue can promote organismal growth via secreted factors (Ghosh et al., 2014); it can equally affect not only cell proliferation but also cell fate decisions in a stem cell lineage (Zhang et al., 2014). Here, we present evidence that Pol I activity itself is a central cause of aging, affecting survival as well as multiple indices of health in the animal model *Drosophila melanogaster*.

## Results

### Pol I Activity in the Adult Limits Animal Lifespan

**Figure 1:**
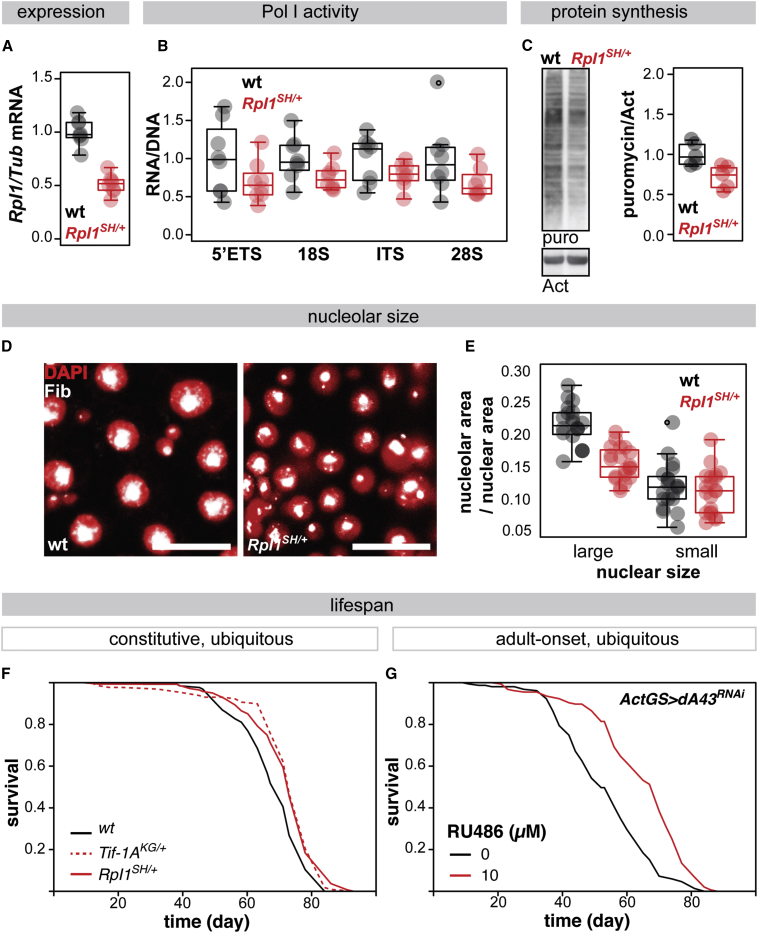
Reduction in Pol I Activity Extends Fruit Fly Lifespan (A) Relative *RpI1* transcript levels (n = 8 biologically independent samples, p < 10^−4^, t test). (B) Ratio of RNA to DNA for the sequences present in pre-rRNA-rDNA (n = 8 biologically independent samples, effect of genotype p = 9 × 10^−4^, no significant effect of the target sequence or interaction, linear model [LM]). (C) Relative levels of protein synthesis in whole flies determined by puromycin incorporation and western blotting, showing a representative blot (left) and quantification (right; n = 5 individual flies, p = 0.01, t test). (D) Representative images of nucleoli (Fibrillarin staining) in posterior midguts (scale bar, 10 μm). (E) Quantification of nucleolar size in large or small nuclei (area as proportion of nuclear area; n = 1–5 nucleoli per animal per nuclear size, 4–5 animals where values from the same animal are indicated as vertically aligned points in the boxplot, effect of genotype p = 0.01, nuclear size p < 1 × 10^−4^, interaction p = 2 × 10^−4^, mixed-effects LM with “animal” as random effect). (F) Lifespans of *RpI1*^*SH/+*^ (n = 137 dead/15 censored flies), *Tif-1A*^*KG/+*^ (n = 118 dead/25 censored flies) and wild-type females (n = 118 dead/22 censored flies, p = 3 × 10^−4^ and 1 × 10^−4^, respectively, log-rank test). (G) Lifespans of females after ubiquitous induction of *dA43*^*RNAi*^ from day 2 of adulthood by RU486 feeding and controls (−RU486 n = 154 dead/4 censored flies, +RU486 n = 155 dead/1 censored flies, p = 4 × 10^−12^, log-rank test). (A)–(E) were assessed in RpI1^SH/+^ and wild-type females. Boxplots show means and quintiles, with individual replicate points overlaid. See also Figures S1 and S2.

Pol I is composed of 14 subunits, several of which are specific to the enzyme (Vannini and Cramer, 2012, Fernández-Tornero et al., 2013, Engel et al., 2013). To examine aging in a Pol I loss-of-function mutant, we backcrossed a transposon insertion in the 5′ region of the *Rpl1* gene (P-element, SH0507) encoding the largest specific subunit of Pol I (ortholog of the A190 subunit of budding yeast [Figure S1A] and the human POLR1A) into a healthy, outbred, wild-type fly background. The *RpI1*^*SH0507*^ allele (*RpI1*^*SH*^ henceforth) is recessive, pre-adult lethal. The viable *RpI1*^*SH/+*^ adult heterozygote females had a 50% reduction in *RpI1* mRNA (p < 10^−4^; Figure 1A), while not showing a significant effect on the expression of the neighboring gene *Blos1* (p = 0.13; Figure S1B), confirming a substantial and specific effect of the insertion on the expression of *RpI1*. Pol I activity is essential for animal growth, which in *Drosophila* occurs during the larval stages (Grewal et al., 2007). *RpI1*^*SH/+*^ females did not weigh substantially less than controls (Figure S1C), indicating that the heterozygous mutation does not impose a major growth impairment.

To examine whether the *RpI1*^*SH/+*^ adults exhibit phenotypes consistent with partial Pol I inactivation, we examined Pol I activity, rates of protein synthesis, and nucleolar size in the mutants. To characterize Pol I activity, we determined the relative RNA to DNA abundance of sequences present in the pre-rRNA—within the 5′ externally transcribed spacer (5′ETS), internally transcribed spacer (ITS), and the mature 18S and 28S rRNA. A 30% overall reduction in this rRNA/rDNA ratio was observed in *RpI1*^*SH/+*^ adult females (p = 9 × 10^−^^4^ Figure 1B), confirming a partial loss of Pol I activity *in vivo*. Using puromycin incorporation assays,we found that this reduction in rRNA synthesis was accompanied by a reduction in the rates of protein synthesis in individual,whole *RpI1*^*SH/+*^ females (p = 0.01; Figures 1C and S1D).

Pre-rRNA transcription and ribosome biogenesis take place in the nucleolus, a subnuclear compartment whose formation is seeded by the rDNA repeats (Grummt, 2003). Nucleolar size is indicative of the levels of ribosome biogenesis (Tiku and Antebi, 2018, Tiku et al., 2017, Uppaluri et al., 2016). To investigate the cellular consequences of the reduction in Pol I activity in *RpI1*^*SH/+*^ females, we examined the nucleolar size of the *Drosophila* midgut (equivalent to the mammalian small intestine). This organ harbors cells with a large nucleus, the absorptive, post-mitotic enterocytes (ECs), and two types of cells with smaller nuclei, the enteroendocrine cells and the mitotically active intestinal stem cells (ISCs) (Miguel-Aliaga et al., 2018). We assessed whether the relative nucleolar size in gut cells was altered in *RpI1*^*SH/+*^ females by staining for the nucleolar marker Fibrillarin and analyzing the data with a mixed-effects linear model (LM) to account for different nuclear sizes and intraindividual correlation. We found that *RpI1*^*SH/+*^ females displayed a reduction in relative nucleolar area compared to controls (p = 0.01; Figures 1D and 1E). This reduction was greatest in ECs (genotype-by-nuclear size interaction p < 10^−4^; for a summary of the LM analysis, see Figure S1E). This was possibly due to the higher demand for protein synthesis in ECs, as indicated by their larger relative nucleolar size (Figures 1D and 1E). We did not observe a substantial difference in nuclear size or ploidy (measured as DAPI staining intensity) between the genotypes (Figures S1F and S1G), which is consistent with a limited effect of the heterozygote mutation on fly growth (Figure S1C). In summary, a heterozygous loss-of-function mutant in the gene encoding the largest subunit of Pol I is viable and displays molecular and cellular phenotypes that are consistent with a partial reduction in Pol I activity. We next sought to examine how this reduction in Pol I activity affects aging.

To characterize the role of Pol I activity in aging, we focused on females, whose aging is more malleable and better characterized. We found that *RpI1*^*SH/+*^ females lived longer than the wild-type controls (Figure 1F). To determine the statistical significance of this observation, we used the log-rank test, which assesses the difference in survival across the lifespan and is highly sensitive. *RpI1*^*SH/+*^ females were significantly longer lived than the wild-type (p = 3 × 10^−4^). To confirm that this longevity is due to a reduction in Pol I activity, we used additional, independent genetic reagents. We backcrossed the previously characterized *Tif-1A*^*KG06857*^ allele (*Tif-1A*^*KG*^ henceforth), which abolishes almost completely the expression of the essential activator of Pol I, *Tif-1A*, resulting in a reduction in Pol I activity (Grewal et al., 2007). *Tif-1A*^*KG/+*^ females also showed a significant lifespan extension (p = 1 × 10^−4^; Figure 1F). Furthermore, the longevity of both *RpI1*^*SH/+*^ and *Tif-1A*^*KG/+*^ females was robustly observed in three independent experimental trials with an average 8% extension of the median lifespan (Figure S1H). Hence, partial lifelong reduction in Pol I activity extends lifespan.

As Pol I is crucial for fly development (Grewal et al., 2007, Ghosh et al., 2014), the longevity observed in the *RpI1*^*SH/+*^ or *Tif-1A*^*KG/+*^ females could have resulted from a developmental effect of reduced Pol I activity, such as reduced growth, that influenced the subsequent adult lifespan. To examine the consequence of reducing Pol I activity specifically in adulthood, we targeted either *Tif-1A* or the *Drosophila* gene encoding the Pol I-specific subunit A43 (*dA43*, *CG13773*; Figure S1A), with RNA interference (RNAi) in combination with inducible, ubiquitous *Actin-* or *daughterless-GeneSwitch* drivers (*ActGS* or *daGS*, respectively) (Osterwalder et al., 2001). The efficacy of the *Tif-1A*^*RNAi*^ line has been demonstrated (Ghosh et al., 2014), and we further confirmed that the ubiquitous expression of either the *dA43*^*RNAi*^ or *Tif-1A*^*RNAi*^ construct during development resulted in lethality, as expected (Figure S2A). Their ubiquitous induction in adulthood, achieved by feeding *ActGS>dA43*^*RNAi*^ or *daGS>Tif-1A*^*RNAi*^ females with the RU486 inducer from day 2 post-eclosion, was enough to extend lifespan (p = 4 × 10^−12^ for Figure 1G; see also Figures S2B and S2C). These data indicate that the developmental roles of Pol I, such as promoting growth, are separable from the role of Pol I in longevity, as is the case for the growth-promoting insulin/insulin-like growth factor signaling pathway (Clancy et al., 2001). In addition, RU486 feeding did not have a significant effect on the lifespans of the driver- or transgene-alone controls (Figures S2D–S2G). Note that we tested a range of RU486 doses and found that higher doses of the inducer produced a diminished or negative effect (Figures S2B and S2C), indicating that an extensive reduction in Pol I activity may be detrimental. Taken together, these experiments reveal that Pol I activity in the adult limits animal lifespan.

### Pol I Activity in Multiple, Distinct Adult Cell Types Affects Organismal Aging

**Figure 2:**
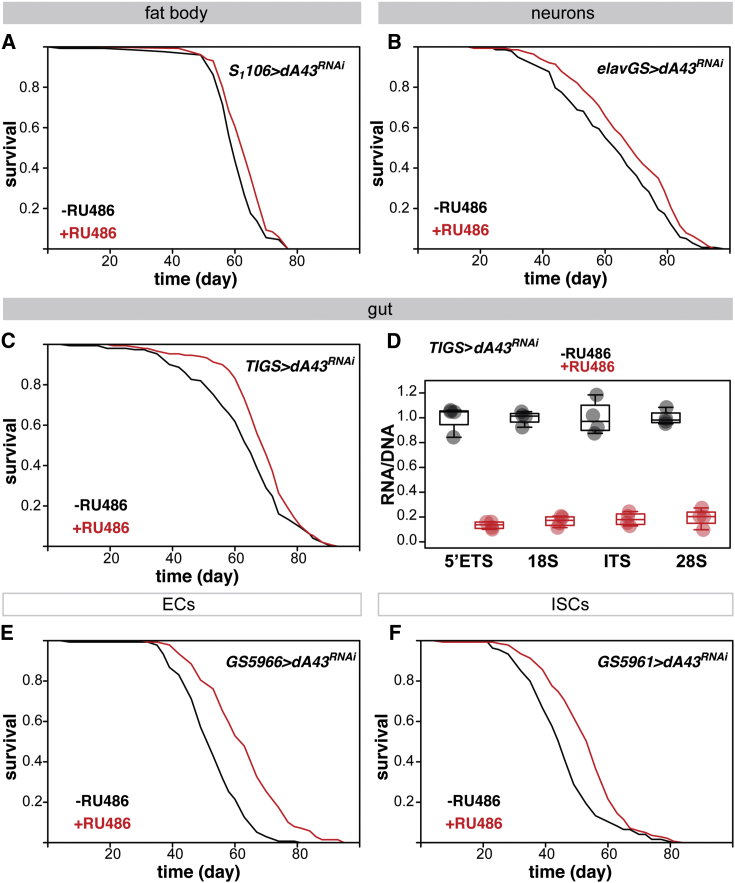
Tissue- and Cell-Type-Restricted Inhibition of Pol I Promotes Longevity (A) Lifespans of females with *dA43*^*RNAi*^ induced in adult fat body and gut by RU486 feeding and controls (−RU486 n = 118 dead/17 censored flies, +RU486 n = 127 dead/12 censored flies, p = 6 × 10^−3^, log-rank test). (B) Lifespan of females with adult-onset induction of *dA43*^*RNAi*^ in neurons achieved by RU486 feeding (−RU486 n = 138 dead/1 censored flies, +RU486 n = 140 dead/1 censored flies, p = 0.01, log-rank test). (C) Lifespans of females with *dA43*^*RNAi*^ induced in the adult gut by RU486 feeding and controls (−RU486 n = 149 dead/4 censored flies, +RU486 n = 148 dead/2 censored flies, p = 7 × 10^−3^, log-rank test). (D) Ratio of RNA to DNA for the sequences present in pre-rRNA-rDNA in fly guts after adult-onset induction of *dA43*^*RNAi*^ (n = 4 biologically independent samples, effect of RU486 p < 10^−4^, no significant effect of the target sequence or interaction, LM). Boxplots show means and quintiles, with individual biological replicate values overlaid as points. (E) Lifespans of females with *dA43*^*RNAi*^ induced in adult ECs by RU486 feeding and controls (−RU486 n = 135 dead/8 censored flies, +RU486 n = 138 dead/9 censored flies, p = 1 × 10^−13^, log-rank test). (F) Lifespans of females with *dA43*^*RNAi*^ induced in adult ISCs by RU486 feeding and controls (−RU486 n = 134 dead/2 censored flies, +RU486 n = 139 dead/5 censored flies, p = 6 × 10^−6^, log-rank test). Fly genotypes are indicated in each panel. See also Figures S2 and S3.

Pol I could limit lifespan from discrete subsets of adult cells. To map where its activity is relevant to longevity, we induced *dA43*^*RNAi*^ or *Tif-1A*^*RNAi*^ constructs using a panel of tissue- or cell-specific GeneSwitch drivers. Driving the *dA43*^*RNAi*^ or *Tif-1A*^*RNAi*^ constructs in the fat body and midgut (the former being equivalent to mammalian adipose tissue and liver) with the *S*_*1*_*106* driver or in neurons with *elavGS* showed modest effects, significantly extending lifespan in 2 of 3 and 1 of 2 experimental trials, respectively (p = 6 × 10^−3^, 2 × 10^−4^, and >0.05; Figures 2A, S3A, and S3B; p = 0.01 and >0.05, Figures 2B and S3C), indicating some, albeit moderate, relevance of these tissues. The induction of *dA43*^*RNAi*^ in the fly muscle with *MHCGS* was detrimental (Figure S3D). This adverse effect may reflect a high requirement for protein synthesis to maintain muscle function. In contrast, knocking down Pol I transcriptional machinery in the midgut with the midgut-restricted *TIGS* driver (Poirier et al., 2008) consistently extended lifespan (p < 0.01; Figures 2C and S3E). We confirmed the expected reduction in the pre-rRNA:rDNA ratio in the midgut of *TIGS>dA43*^*RNAi*^ females (p = 1 × 10^−4^; Figure 2D). Overall, the survey of *Drosophila* tissues indicated that the main longevity effect of Pol I inhibition stems from the midgut, with possible minor contributions from fat body cells and neurons.

The *Drosophila* midgut contains several cell types. Driving *dA43*^*RNAi*^ in the post-mitotic ECs or mitotically active ISCs was sufficient to extend lifespan (*GS5966* and *GS5961* drivers, respectively, p < 10^−5^; Figures 2E and 2F). In all of the cases, RU486 feeding had no effect on the lifespans of driver-alone controls (Figures S3F–S3H). Overall, our data revealed that Pol I activity drives aging from distinct adult cell types. Pol I acts non-redundantly from both post-mitotic cells, such as ECs, and cells with a proliferation potential, the ISCs. We next focused on the ISCs due to their importance in gut homeostasis (Miguel-Aliaga et al., 2018) and the known role of Pol III in these cells (Filer et al., 2017).

### rRNA Biogenesis in the ISCs Limits Lifespan

**Figure 3:**
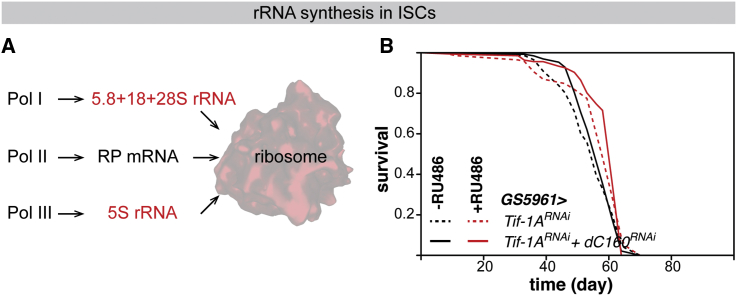
Pol I and Pol III Act in the Same Longevity Pathway in the ISCs (A) Contribution of each Pol to ribosome biogenesis. rRNAs are indicated in red. RP, ribosomal protein. (B) Lifespans of females with *dA43*^*RNAi*^ alone (−RU486 n = 138 dead/6 censored flies, +RU486 n = 137 dead/5 censored flies, p = 0.01, log-rank test) or together with *dC160*^*RNAi*^ (−RU486 n = 99 dead/32 censored flies, +RU486 n = 127 dead/10 censored flies, p = 2 × 10^−5^, log-rank test) induced in adult ISCs by RU486 feeding and controls. The lifespans of the two −RU486 or the two +RU486 conditions are not significantly different. See Figure S4 for *GS5961>dC160*^*RNAi*^ lifespans that were carried out in parallel and the CPH analysis.

Ribosome biogenesis is emerging as an important process in the regulation of stem cell behaviors (Zhang et al., 2014, Sanchez et al., 2016). Reduction in Pol I activity in the ISCs may extend lifespan by limiting the provision of rRNAs required for ribosome biogenesis. While Pol I is the only polymerase that transcribes the rDNA locus, it is not the only polymerase that synthesizes rRNA species; 5S rRNA is generated by Pol III (Figure 3A). We have recently shown that Pol III activity in the gut also limits the lifespan in *Drosophila* (Filer et al., 2017). The fly midgut displays sexually dimorphic physiology and aging (Regan et al., 2016, Hudry et al., 2016, Hudry et al., 2019), and similar to the effects of Pol III, inhibition of Pol I in the midgut did not extend lifespan in males (Figure S4A). A further similarity between the effects of Pol I and Pol III is that each polymerase limits lifespan from the ISCs (see Figure 2F and Filer et al., 2017). This highlights the ISCs as the midgut cells in which both Pol I and Pol III are relevant to lifespan and highlights rRNA synthesis in the ISCs as a mechanism of longevity downstream of Pol I and Pol III, since the one task shared by both Pols is to provide the full, requisite complement of rRNA species (Kressler et al., 2017).

To further examine the connection between rRNA synthesis and longevity, we directly assessed the genetic interactions between Pol I and Pol III activity in the ISCs for lifespan. If the two Pols do not act through a common longevity mechanism, we would expect the effects of reducing their activity on lifespan to be additive. We found that inducing the expression of a validated RNAi line against the largest Pol III subunit, *dC160* (Filer et al., 2017), did not further extend lifespan when co-expressed with *Tif-1A*^*RNAi*^ in the ISCs (Figure 3B), while it did when expressed alone in a parallel experiment (Figure S4B). Note that the presence of UAS-*Tif-1A*^*RNAi*^ did not appear to hamper the ability of the *GS5961* driver to induce a second transgene (Figure S4C). For a robust analysis of survival data, we used a Cox proportional hazards (CPH) model, which can assess the statistical significance of individual effects (genotype and presence of RU486) and of their interaction to determine whether the lifespan response to RU486 is altered by the presence of both RNAi lines. CPH analysis confirmed that the lifespan extension obtained by reducing Pol I or Pol III activity individually in the ISCs was not significantly different from that observed when both were targeted simultaneously (Figure S4D). This result is consistent with Pol I and Pol III acting in the same longevity pathway in the ISCs. This conclusion is additionally supported by the known roles of the two polymerases in the same process, namely ribosome biogenesis, and the similar profile of organs whence their activity affects lifespan. While it may be tempting to view one Pol as upstream of the other, such a cascade is unlikely since the three nuclear polymerases appear tightly coordinated for ribosome biogenesis (Martin et al., 2006, Filer et al., 2017, Grummt, 2003).

### Pol I Activity Defines a Central Aging Process with Broad Effects on Health

Having established that a moderate reduction in Pol I activity was sufficient to extend lifespan, we next sought to examine how Pol I activity affects health in old age. Since the midgut appeared critical for longevity, we initially focused on the health and integrity of this organ.

**Figure 4:**
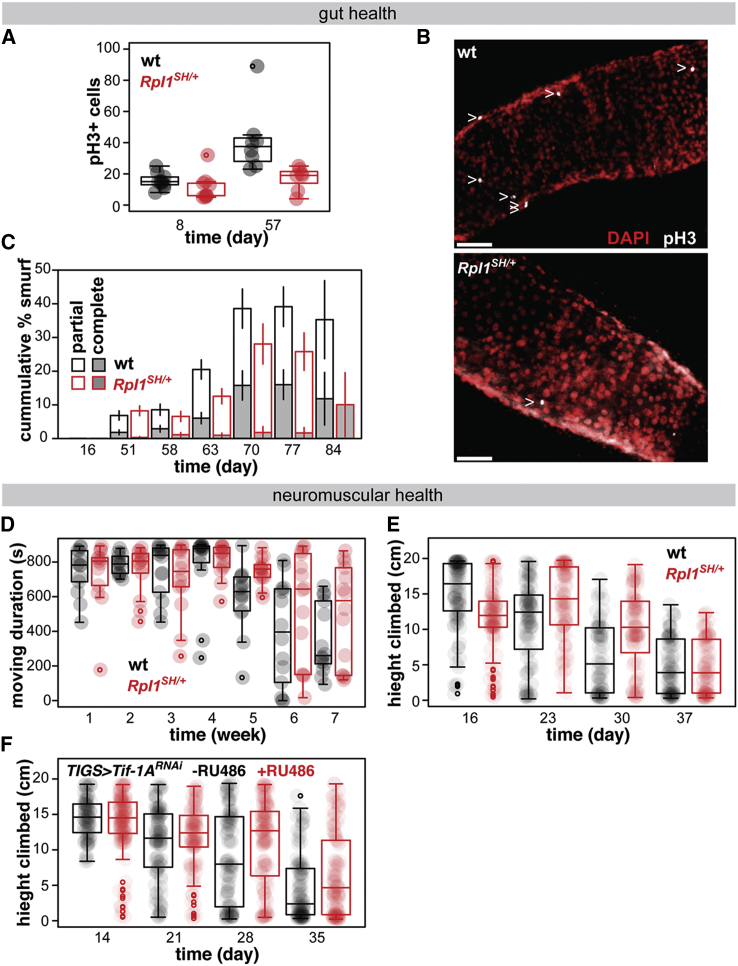
Reduction in Pol I Activity Improves Gut Health in Old Flies (A) Number of pH3^+^ cells per gut (n = 8–10 flies, effect of age p = 6 × 10^−4^, genotype p = 3 × 10^−3^, genotype-by-age interaction p = 0.02, LM). Boxplots show means and quintiles, with counts from individual guts overlaid as points. (B) Representative images of pH3^+^ cells in old guts (white > indicates pH3^+^ cells, stacks; scale bar, 50 μm). (C) Cumulative proportion of partial and complete smurfs (means ± SEs, n = 10–378 flies, no significant effect of genotype, age p < 10^−4^, genotype-by-age interaction p < 0.05, ordinal logistic regression). (D) Moving duration during exploratory walking in *RpI1*^*SH/+*^ and wild-type females (n = 13–14 flies, effect of genotype not significant, age p < 10^−4^, genotype-by-age interaction p = 0.01, LM). (E) Height climbed during negative geotaxis assays in *RpI1*^*SH/+*^ and wild-type females (n = 79–97 flies, no significant effect of genotype, age p < 10^−4^, genotype-by-age interaction p = 1 × 10^−4^, LM). (F) Height climbed during negative geotaxis assays in *TIGS>Tif-1A*^*RNAi*^ females in the presence or absence of RU486 (n = 90–123 flies, effect of RU486 p = 2.5 × 10^−3^, age p < 10^−4^, genotype-by-age interaction p = 4.8 × 10^−3^, LM). (A)–(E) were assessed in RpI1^SH/+^ and wild-type females. Boxplots show means and quintiles, with values for individual flies overlaid as points. See also Figure S4.

As *Drosophila* females age, the number of mitotic cells in the midgut increases due to ISC hyperproliferation and misdifferentiation (Biteau et al., 2008, Miguel-Aliaga et al., 2018). This age-induced hyperplasia can be monitored by scoring the number of cells harboring phosphorylated histone H3 (pH3). We examined the number of pH3^+^ cells in young and old *RpI1*^*SH/+*^ females. As for all age-related phenotypes, we analyzed the data using an LM to determine the impact of the mutation on age-related changes (assessed by the significance of the age-by-genotype interaction). We found that *RpI1*^*SH/+*^ females displayed a reduction in age-related hyperplasia in the gut, relative to wild type (p = 0.02 for age-by-genotype interaction; Figures 4A and 4B). Note that the pronounced difference in the number of dividing cells between wild-type and mutant females was specifically observed in older flies (Figure 4A), indicating that it is due to the prevention of a pathology, rather than a substantial alteration of ISC function under homeostatic conditions.

In addition and linked to this hyperproliferative phenotype, the ability of the fly gut to act as a barrier becomes compromised during aging (Rera et al., 2012, Clark et al., 2015, Miguel-Aliaga et al., 2018). The age-related loss of gut barrier function can be measured as the ability to exclude an orally administered dye from the body cavity. We performed the previously described “smurf” assays (Rera et al., 2012) and found that this loss of gut barrier function was delayed in *RpI1*^*SH/+*^ females relative to controls (p < 0.05 for age-by-genotype interaction; Figure 4C). Hence, a moderate reduction in Pol I activity delays multiple, interlinked aspects of intestinal aging.

In humans, age is the main risk factor for a number of diseases and dysfunctions affecting a range of organ systems. Such manifestations of aging and extensive experimental observations imply that there are common etiologies for a number of age-related diseases (Gems, 2015, Partridge et al., 2018). To examine whether Pol I activity is one such central driver of multiple manifestations of aging, we next examined the age-related changes in the performance of the neuromuscular system in *RpI1*^*SH/+*^ and wild-type females.

When placed into a new environment, flies display a complex, spontaneous locomotor behavior called exploratory walking. The performance of this behavior is negatively influenced by age and is indicative of declining brain and locomotor function (Ismail et al., 2015). We characterized the walking behavior of *RpI1*^*SH/+*^ and wild-type females over 7 weeks. In *RpI1*^*SH/+*^ females, age-related changes in some parameters of exploratory walking were delayed relative to wild type, and specifically, the age-induced decrease in the time spent walking (walking duration) was significantly rescued in the heterozygous mutants (p = 0.01 for age-by-genotype interaction; Figure 4D). While not all of the parameters were significantly improved (Figures S4E–S4H), the observed beneficial effect on walking duration indicates a better preservation of aspects of brain function in aged *RpI1*^*SH/+*^ females.

We next examined negative geotaxis as a complementary assay of neuromuscular function. This is an induced escape response that also diminishes with age and can be measured as the ability to climb a vertical surface (Gargano et al., 2005). The age-related decline in climbing ability was delayed in *RpI1*^*SH/+*^ females relative to wild type (p = 1 × 10^−4^ for age-by-genotype interaction; Figure 4E). This delay could also be observed upon knock down of *Tif-1A* specifically in the adult midgut (p = 4.8 × 10^−3^ for age-by-genotype interaction; Figure 4F), indicating that Pol I activity in one organ can affect the health of another. Since the decline in climbing ability appears to precede any obvious gut pathology (compare Figures 4A–4C with 4D–4F), it is unlikely that the rescue of age-related gut pathology directly causes an improvement in climbing. Overall, *RpI1*^*SH/+*^ females appear to better maintain their neuromuscular system with age. Considering this together with the observed improvements in age-related gut function, Pol I activity emerges as a common driver of several, apparently unrelated, age-induced deficits in multiple organ systems.

## Discussion

Previous studies have linked eukaryotic aging to the rDNA locus (Tiku and Antebi, 2018). rDNA integrity and stability have been causally implicated in replicative aging in yeast (Sinclair and Guarente, 1997, Kaeberlein et al., 1999, Defossez et al., 1999). More recently, small nucleolar size and reduced expression from the rDNA locus have been highlighted as a common feature of numerous distinct long-lived models from a range of animal species (Tiku et al., 2017, Tiku and Antebi, 2018). In old mice, the nucleoli of hematopoietic stem cells accumulate persistent markers of replication stress that may impede rDNA transcription (Flach et al., 2014), while rDNA copies are lost in aging male fly germline stem cells (Lu et al., 2018). In addition, mouse rDNA may be the site of epigenetic regulation that allows early-life nutrition to program adult physiology and health (Holland et al., 2016). While rDNA and the nucleolus have been linked to aging in several contexts, the role of Pol I has not been thoroughly assessed. In yeast, extrachromosomal rDNA circles accumulate during aging and have recently been reported to drive an excessive rise in Pol I activity, which appears to impair nuclear homeostasis (Morlot et al., 2019). Our study demonstrates that the activity of Pol I itself contributes to aging in the fruit fly. Hence, our findings shed a new light on previous observations linking aging to the rDNA locus, nucleolar structure, and nucleolar function, suggesting that they may be causally connected by Pol I activity. The relevance of this finding is further highlighted by the possibility of the pharmacological inhibition of Pol I activity, with several inhibitors developed (for examples, see Drygin et al., 2010, Wei et al., 2018) that may be harnessed for improved health in older ages.

We present evidence that in the fly ISCs, Pol I acts on animal aging though rRNA biogenesis, a fundamental cellular process that is likely to alter stem cell behaviors in diverse settings (Zhang et al., 2014, Sanchez et al., 2016). Interestingly, this also implicates 5S rRNA biogenesis as a longevity-limiting process downstream of Pol III in the ISCs. Both inhibition of Pol III and its activation have been reported to extend lifespan (Filer et al., 2017, Bonhoure et al., 2015). It is possible that Pol III inhibition achieves its health benefits through limiting 5S rRNA synthesis, while activation may be beneficial due to the metabolic effects of futile tRNA cycling (Willis et al., 2018). Furthermore, the two interventions may be acting from different adult cell types, with Pol III inhibition beneficial in stem cells and activation in differentiated cells, for example.

While rRNA biogenesis appears to be the key process for lifespan in the ISCs, additional mechanisms underlying longevity resulting from Pol I inhibition cannot be excluded in this or other cell types. For example, high levels of transcription of the rDNA locus are thought to make it vulnerable to DNA damage (Tiku and Antebi, 2018, Takeuchi et al., 2003). Reducing Pol I activity may reduce the susceptibility of this locus to age-related DNA damage and instability; Pol I activity is also required for the correct assembly of the nucleolus (Falahati et al., 2016) and may affect a range of nucleolar functions. Indeed, processes other than rRNA biogenesis, such as genomic instability or altered nucleolar function, are likely to be important in at least some adult cell types in which Pol I activity is a determinant of aging.

In addition, Pol I may be relevant in many contexts. Pol I activity is essential for cell growth and proliferation; the growth- and proliferation-simulating PI3K-AKT-TOR and Ras-RAF-ERK signaling pathways, as well as the transcription factor Myc, converge to activate Pol I transcription (Kusnadi et al., 2015). The inhibition of each of these Pol I activators is now known to extend animal lifespans (López-Otín et al., 2013, Slack et al., 2015, Greer et al., 2013, Harrison et al., 2009, Hofmann et al., 2015), indicating that, similar to Pol I, their activity limits the lifespan of the wild-type animal. It is tempting to speculate that these longevity interventions act in part by reducing Pol I activity and that Pol I bridges between these pathways and the rDNA locus. Furthermore, consistent with the antagonistic pleiotropy theory of the evolution of lifespan (Williams, 1957), it is likely that Pol I activity in the wild-type animal is set at levels that are required for development and reproduction at the expense of later life health and survival.

## STAR★Methods

### Key Resources Table

**Table:** 

REAGENT or RESOURCE	SOURCE	IDENTIFIER
**Antibodies**		

Mouse polyclonal Anti-Fibrillarin antibody	Abcam	Cat. #ab5821; RRID:AB_2105785
Rabbit polyclonal Phospho-Histone H3 (Ser10) Antibody	Cell Signaling	Cat. #9701; RRID:AB_331535
GFP Polyclonal Antibody, Alexa Fluor 488	Thermo Fisher Scientific	Cat. # A-21311; RRID:AB_221477
Alexa Fluor 594 Donkey anti-Rabbit IgG H&L Secondary Antibody	Thermo Fisher Scientific	Cat. A-21207; RRID:AB_141637
Alexa Fluor 488 Donkey anti-Mouse IgG H&L Secondary Antibody	Thermo Fisher Scientific	Cat. A-21202; RRID:AB_141607
Anti-beta Actin antibody	Abcam	Cat. #8224; RRID:AB_449644
Anti-Puromycin antibody, clone 12D10	Millipore	Cat. #MABE343MI; RRID:AB_2566826
Goat Anti-Mouse IgG H&L (HRP) Antibody	Abcam	Cat. # ab6789; RRID:AB_955439

**Chemicals, Peptides, and Recombinant Proteins**		

FD&C Blue dye No.1	Fastcolors	CI: 42090 CAS Number: 3844-45-9
Vectashield with DAPI	Vector Laboratories	Cat# H-1200; RRID:AB_2336790
TRIzol Reagent	Thermo Fisher Scientific	Cat. #15596026
Schneider’s *Drosophila* medium	Sigma	# S0146
RU^486^	Sigma	#M8046
Puromycin	GIBCO	#A1113803

**Critical Commercial Assays**		

NuPAGE 4-12% Bis-Tris Protein Gel	Invitrogen	#NP0322BOX
SYBR Green PCR Master Mix	Applied Biosystems	Cat. # 4309155
SuperScript II Reverse Transcriptase	Thermo Fisher Scientifc	Cat. #18064014

**Experimental Models: Organisms/Strains**		

*D. melanogaster*: w^Dah^	This lab / Linda Partridge	N/A
*D. melanogaster: w*^*1118*^	*Bloomington Drosophila Stock Centre*	RRID:BDSC_3605
*D. melanogaster* MSCGS driver	Poirier et al., 2008	N/A
*D. melanogaster* TIGS driver	Poirier et al., 2008	N/A
*D. melanogaster* S_1_106 driver	Poirier et al., 2008	N/A
*D. melanogaster* GS5961 driver	Mathur et al., 2010	N/A
*D. melanogaster* GS5966 driver	Mathur et al., 2010	N/A
*elavGS*	Niccoli et al., 2016, Tricoire et al., 2009	N/A
*daughterlessGeneSwitch*	Tricoire et al., 2009	N/A
*ActinGeneSwitch*	Alic et al., 2011, Ford et al., 2007	N/A
*daughterlessGAL4*	Bloomington Drosophila Stock Centre	RRID:BDSC_55850
*RpI1*^*SH0507*^	*Bloomington Drosophila Stock Centre*	RRID:BDSC_29480
*Tif-1A*^*KG06857*^	*Bloomington Drosophila Stock Centre*	RRID:BDSC_14507
*UAS-dA43*^*RNAi*^	Vienna Drosophila Resource Center	v103392
*UAS-Tif-1A*^*RNAi*^	Vienna Drosophila Resource Center	v20334
*UAS-dC160*^*RNAi*^	Vienna Drosophila Resource Center	v30512
*UAS-CD8-GFP*	Linda Partridge	N/A

**Oligonucleotides**		

*Drosophila RpI1*-F 5′ TAAGCTTCCGCCCTCGCCAC 3′	Eurofins	N/A
*Drosophila RpI1*-R 5′ TAACCGACAGCCCTCGCTGC 3′	Eurofins	N/A
*Drosophila Blos1*-F 5′ GCGAAAACAGGAACAGGAGG 3′	Eurofins	N/A
*Drosophila Blos1*-R 5′ GTCCAGCCGCTTCTGGTTC 3′	Eurofins	N/A
pre-rRNA ITS-F 5′ TTAGTGTGGGGCTTGGCAACCT 3′	Eurofins	N/A
pre-rRNA ITS-R 5′ CGCCGTTGTTGTAAGTACTCGCC 3′	Eurofins	N/A
pre-rRNA ETS-F 5′ GTTGCCGACCTCGCATTGTTCG 3′	Eurofins	N/A
pre-rRNA ETS-R 5′ CGGAGCCAAGTCCCGTGTTCAA 3′	Eurofins	N/A
pre-rRNA 18S-F 5′ TGTAGCCTTCATTCATGTTGGCAG 3′	Eurofins	N/A
pre-rRNA 18S-R 5′ ACCAACAGGTACGGCTCCAC 3′	Eurofins	N/A
pre-rRNA 28S-F 5′ CCTGCCGAAGCAACTAGCCCTT 3′	Eurofins	N/A
pre-rRNA 28S-R 5′ CCATGCAGGCTTACGCCAAAC 3′	Eurofins	N/A
*Drosophila Tubulin*-F 5′ TGGGCCCGTCTGGACCACAA 3′	Eurofins	N/A
*Drosophila Tubulin*-R 5′ TCGCCGTCACCGGAGTCCAT 3′	Eurofins	N/A

**Software and Algorithms**		

Microsoft Excel	Microsoft	https://www.microsoft.com/en-gb/
Fiji	ImageJ	(Schindelin et al., 2012)
R statistics package	R Core Team	https://www.r-project.org/
JMP13	SAS	https://www.jmp.com/en_be/home.html
Ethovision XT video tracking software	Noldus	https://www.noldus.com/ethovision-xt
Adobe Photoshop	Adobe	https://www.adobe.com/uk/products/photoshop.html?promoid=PC1PQQ5T&mv=other
Zeiss Zen	Zeiss	https://www.zeiss.com/microscopy/int/products/microscope-software/zen.html

**Other**		

Drosoflippers	Drosoflipper	www.drosoflipper.com
Zeiss LSM 700 confocal laser scanning microscope	Zeiss	N/A
QuantStudio 6cFlex real-time PCR machine	Thermo Fisher Scientific	N/A
Nanodrop 2000C spectrophotometer	Thermo Fisher Scientific	N/A
Glass beads	Sigma	#G8772
Mettler Toledo AT201 precision balance	Mettler Toledo	N/A

### Lead Contact and Materials Availability

Further information and requests for resources and reagents should be directed and will be fulfilled by the Lead Contact, Nazif Alic (n.alic@ucl.ac.uk). This study did not generate new unique reagents.

### Experimental Model and Subject Details

The outbred, wild-type stock was collected in 1970 in Dahomey (now Benin) and has been kept in population cages to maintain lifespan and fecundity at levels similar to wild-caught flies. The white Dahomey (w^Dah^) stock was derived by incorporation of the *w*^*1118*^ mutation into the outbred Dahomey background by successive backcrossing and Wolbachia-cleared by tetracycline treatment. All mutants and transgenes were backcrossed at least six times into this background. Where required, combinations of transgenes were generated using standard fly genetic approaches while avoiding population bottlenecks. Stocks were maintained and experiments conducted at 25°C on a 12L:12D cycle at 60% humidity, on SYA food (Bass et al., 2007) containing 10% brewer’s yeast, 5% sucrose, and 1.5% agar (all w/v). Experiments were performed on females (except the experiment shown in Figure S4A, which was done on males) housed 10-15 per vial. The researchers were not blinded to the conditions.

### Method Details

#### Fly husbandry and lifespan assays

Experimental flies were generated from suitable crosses, eggs collected over a 22-24h period, recovered in PBS and 18-20 μL of egg sediment was placed in bottles containing SYA medium to rear flies at standardized larval density. Once emerged, the adults were transferred to fresh bottles, allowed to mate for 48 h and females sorted into experimental vials at a density of 15 flies per vial (10 flies per vial for RNA extractions). Where required, RU486 (Sigma #M8046, dissolved in ethanol) was added to 200 μM final concentration or as indicated. For control treatments, the volume of the vehicle alone corresponding to the highest concentration was added. Note that for exploratory walking assays, the relevant mutants or wild-type were crossed into Dahomey (Wolbachia negative) so that all experimental flies were *w/w*^*1118*^. For lifespan assays, flies were transferred to fresh vials and their survival was scored two-three times a week.

#### Weight measurements

To measure fly body weight, individual adult female flies were snap-frozen in liquid nitrogen and weighed on a Mettler Toledo AT201 precision balance (analytical weighing to within 0.0001 g).

#### Exploratory walking assays

Exploratory walking assay has been described (Ismail et al., 2015, Martin, 2004). At indicated times, individual flies were placed in 4cm diameter/1cm height circular Perspex arena, allowed to rest for one minute and then were video recorded for 15 minutes. Videos were analyzed using Ethovision XT video tracking software (Noldus) to extract the parameters of each walk. Naive flies were used at each time point.

#### Negative geotaxis assays

Flies were tipped using *Drosoflippers* (www.drosoflipper.com). At indicated times, flies were transferred to empty vials placed so that they could climb 2 vial heights. Flies were allowed to acclimatise for 5 min, gently tipped to the bottom of the vial and climbing was video recorded for 40 s. Video stills from the same time point (15 s; the time point when young wild-type flies start reaching maximum height) were analyzed in Fiji (Schindelin et al., 2012) and coordinates exported to Excel (Microsoft). The same cohort was continuously assayed.

#### Smurf assays

Smurf assays were essentially performed as described (Rera et al., 2012). At the indicated age the flies were placed on SYA food containing 2.5% (w/v) blue dye (FD&C blue dye no. 1, Fastcolors) for 48 h and scored as full smurfs if completely blue or partial smurfs if the dye had leaked out of the gut but not reached the head. The same cohort was continuously assayed.

#### Immunocytochemistry

Midguts were dissected from 7-day-old flies in ice-cold PBS and immediately fixed in 4% formaldehyde for 20-30 min. Guts were washed in PBST (0.2% Triton-X in PBS), blocked with 5% bovine serum albumin or donkey serum in PBST, incubated in primary antibody overnight at 4 °C and in secondary for 2 hr at RT, with washes as required. Guts were mounted in mounting medium containing DAPI (Vector Laboratories). The following antibodies were used for staining: Anti-fibrillarin (1:200, Abcam ab5821) was used to stain the nucleoli, anti-phospho-Histone H3 (1:100, Cell Signaling UK #9701) to stain mitotic cells, and 488-conjugated Alexa Fluor anti-GFP (1/500, Thermo Fisher Scientific #A21311) to mark GFP. The secondary antibodies Alexa Fluor 594 donkey anti-rabbit (Thermo Fisher Scientific #A21207) and Alexa Fluor 488 donkey anti-mouse (Thermo Fisher Scientific, #A21202) were used at a concentration of 1:1000.

#### PH3 quantification, nucleolar measurements, and DAPI intensity quantification

All microscope and representative images (stacks) were taken with a Zeiss LSM700 confocal microscope, unless otherwise stated. PH3 positive cells per midgut were counted on a fluorescence microscope. Fibrillarin staining was imaged and the nucleus (DAPI) was manually traced and measured using Zeiss Zen® software from a random selection of enterocyte nuclei imaged at their widest diameter in the posterior midgut. Images were processed in Fiji (Schindelin et al., 2012) and Adobe Photoshop (Adobe) for publication. To calculate relative DAPI intensity of gut nuclei, adult female flies were dissected and fixed as described above, then mounted in fluorescent medium containing DAPI. DAPI intensity was quantified in every nucleus (traced automatically) and corrected by subtracting the background intensity using Fiji® (Schindelin et al., 2012). DAPI intensity data were expressed relative to the control imaged at the same time.

#### RNA, DNA extraction and qPCR

Total RNA and DNA were quantitatively isolated from the same sample of ten whole 7-day-old adult flies, or ten dissected midguts, using TRIZOL (Invitrogen) according to manufacturer’s instructions. RNA was converted to cDNA using random hexamers and Superscript II (Invitrogen) according to manufacturer’s instructions. Quantitative PCR on cDNA or DNA was performed using Power SYBR Green PCR Master Mix (ABI), on QuantStudio6 Flex real-time PCR, and quantities of each sequence were determined with the relative standard curve method (Larionov et al., 2005). RNA/DNA (or RNA/RNA) ratio was calculated per sample. For gut extractions, data were scaled to batch. Data were expressed relative to the control.

#### Assessing developmental lethality

To assess developmental lethality, development was tracked over 10 d after 2-4 h egg laying period.

#### Puromycin incorporation assays

Thorax and abdomen of individual flies were opened along the ventral midline in ice-cold PBS, with wild-type and mutant flies processed in batches carried out in parallel. Flies were then place in 0.2 mL of ice-cold Schneider’s medium (Sigma, #S0146). 0.8 mL of the same medium containing puromycin (GIBCO, #A1113803) and pre-warmed to 25°C was added and puromycin incorporation was assayed as before (Filer et al., 2017), with the exception that the flies were broken with glass beads (Sigma, #G8772) and protein separated on gradient gels (Thermo Fisher Scientific, #NP0322BOX). The western blotting used anti-puromycin antibody (Milipore, #12D10) with Anti-Actin (Abcam, #ab8224) as the loading control. The intensity of anti-puromycin staining between 15 and 165 kDa was quantified from chemiluminescence images (using an Anti-Mouse secondary HRP antibody, Abcam, #ab6789) in Fiji (Schindelin et al., 2012), relative to Actin, scaled to replicate batch and expressed relative to the control.

### Quantification and Statistical Analysis

Statistical analysis was performed in Excel (Microsoft; long-rank test), JMP-13 (SAS; log-rank test, linear model, mixed effects linear model, ordinal logistic regression, t test) or R (R core team; Cox Proportional Hazards). Where relevant, time was included as a continuous variable. All models had a full factorial design. P values < 0.05 were considered as significant. Details of tests, including the number and nature of replicate samples (n), are given in figure legends and in the text.

### Data Code and Availability

This study did not generate any datasets. Source data for figures is available as a Mendeley dataset https://dx.doi.org/10.17632/46cj62735f.1. All other data will be made available from the corresponding author on request.

## Supplemental Information

Document S1. Figures S1–S4

Document S2. Article plus Supplemental Information
