# Supplementary material for: Partial Inhibition of RNA Polymerase I Promotes Animal Health and Longevity
Source: Cell Rep. 2020 Feb 11;30(6):1661–1669.e4. doi: 10.1016/j.celrep.2020.01.017 (PMC7013379; doi:10.1016/j.celrep.2020.01.017)
Supplement: Document S1. Figures S1–S4 [file mmc1.pdf]

Cell Reports, Volume *30*

Supplemental Information

Partial Inhibition of RNA Polymerase I Promotes Animal Health and Longevity

Guillermo Martínez Corrales, Danny Filer, Katharina C. Wenz, Abbie Rogan, George Phillips, Mengjia Li, Yodit Feseha, Susan J. Broughton, and Nazif Alic

A

yeast subunit	yeast gene	fly gene
A190	*RPA190*	*Rpl1*
A135	*RPA135*	*Rpl135*
A49	*RPA49*	-
A43	*RPA43*	*CG13773*
A34.5	*RPA34*	-
A14	*RPA14*	-
A12.2	*RPA12*	*Rpl12*

B

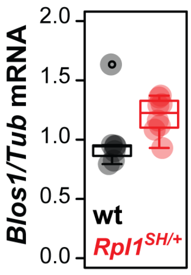

C

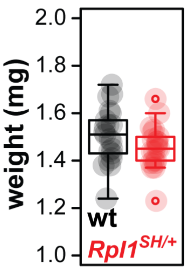

D

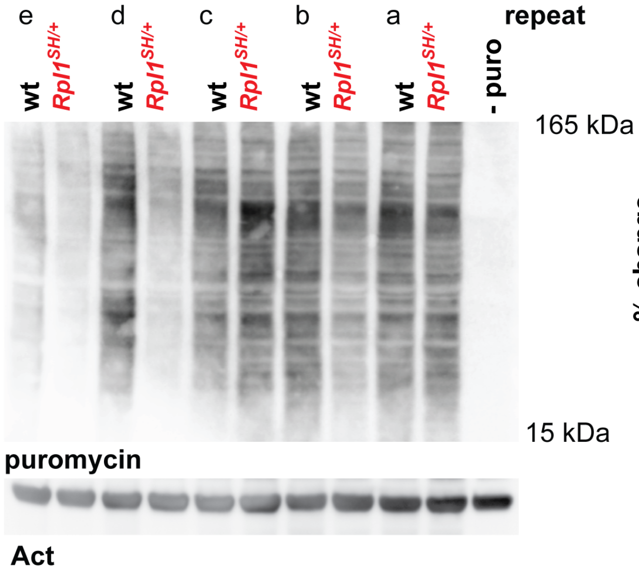

H

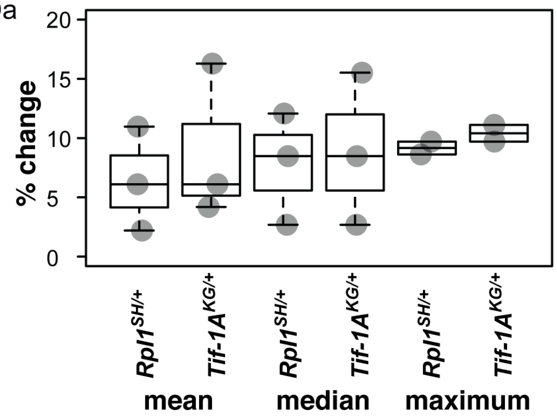

E

covariate	effect	p
intercept	0.151	<1x10-4
genotype (*Rpl1*$^{SH/+}$)	-0.017	0.012
nuclear size (large)	0.035	<1x10-4
genotype (*Rpl1*$^{SH/+}$):nuclear size (large)	-0.014	2x10-4

F

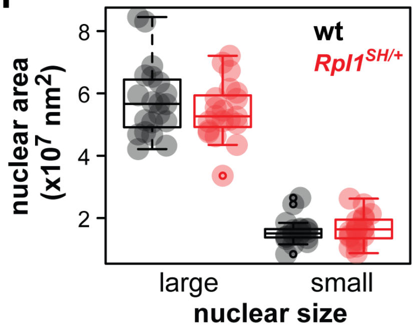

G

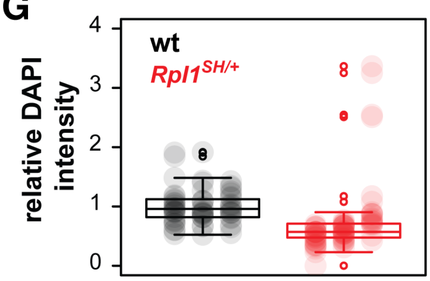

Figure S1 Reduction in Pol I activity extends lifespan. Related to Figure 1.

(A) The fly genes encoding Pol I-specific subunits were identified with BLAST and reverse BLAST using the budding yeast genes. "-" indicates that the subunit could not be identified.

(B) Relative levels of *Blos1* mRNA in *Rpl1*$^{SH/+}$ and wild-type females (n=7-8 biologically independent samples, $p>0.05$, *t-test*).

(C) Weight of *Rpl1*$^{SH/+}$ and wild-type females. *Rpl1*$^{SH/+}$ females tended to weight slightly less than the wild-type but this difference was not significant (n=21 individual flies, $p>0.05$, *t-test*).

(D) Western blot showing all the repeats of the puromycin incorporation assay.

(E) Results of the *mixed effect LM* analysis of relative nucleolar size.

(F) Nuclear size in *Rpl1*$^{SH/+}$ and wild-type female guts (n= 1-5 nuclei per animal per nuclear size, 4-5 animals where values from the same animal are indicated as vertically aligned points in the box plot, effect of genotype $p>0.05$, nuclear size $p<1x10^{-4}$, interaction $p>0.05$, *mixed effects LM* with "animal" as random effect).

(G) DAPI intensities measured in ECs of *Rpl1*$^{SH/+}$ and wild-type females. *Rpl1*$^{SH/+}$ females tended to show lower DAPI signal in ECs than the wild-type but this difference was not significant (n= 20-25 nuclei, 3 animals where values from the same animal are indicated as vertically aligned points in the box plot, effect of genotype $p>0.05$, *mixed effects LM* with "animal" as random effect).

(H) Summary of mean, median and maximum (last 5% surviving) lifespan extension in *Rpl1*$^{SH/+}$ females relative to wild-type females observed in three independent replicates. Lifespan was significantly extended in each ($p<0.05$, *log-rank test*). One of the replicates is shown in Figure 1. Note that due to the pattern of death, the maximum lifespan parameter could not be obtained from one replicate.

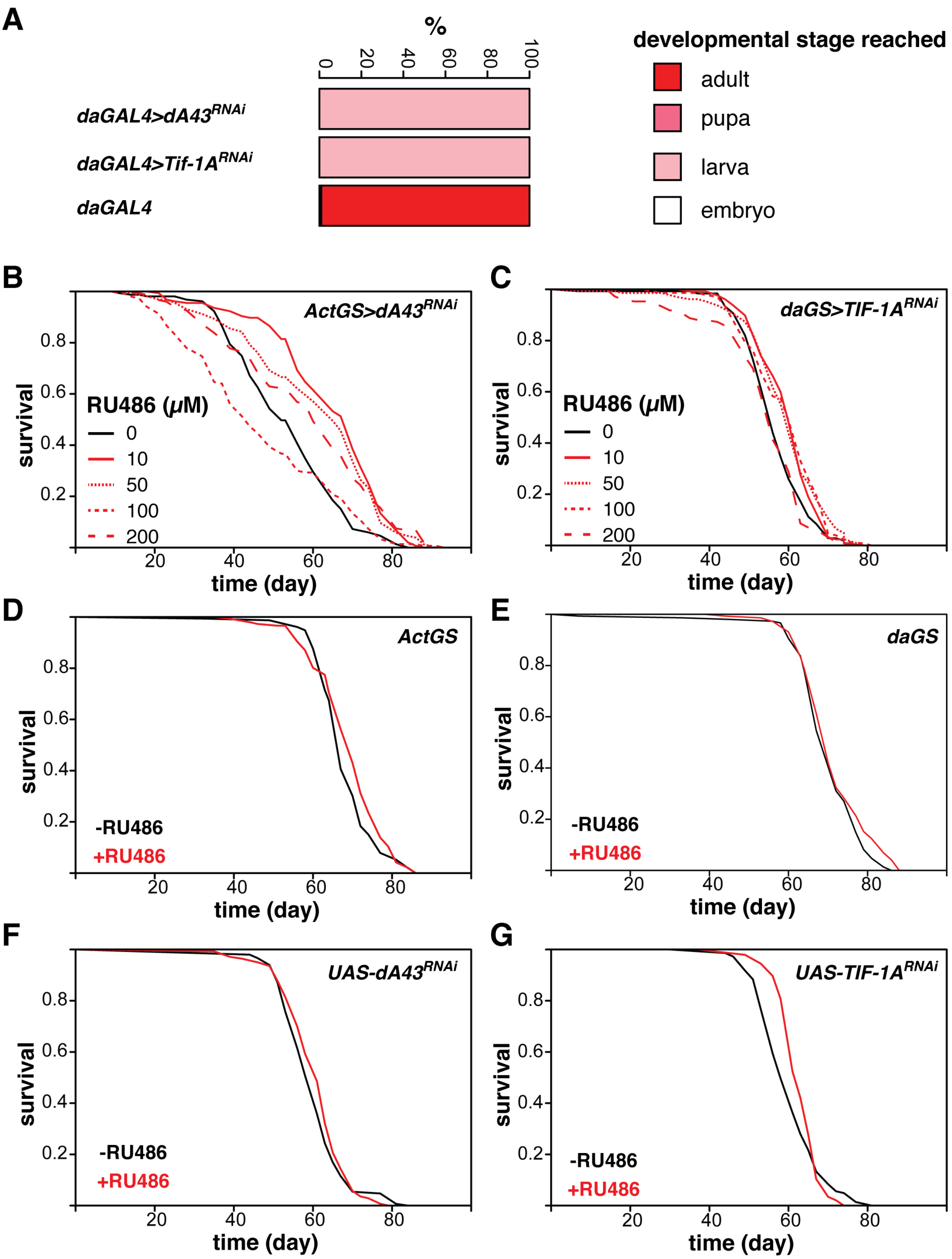
A
%
developmental stage reached
adult
pupa
larva
embryo
daGAL4>dA43RNAi
daGAL4>Tif-1ARNAi
daGAL4
B
ActGS>dA43RNAi
RU486 (μM)
0
10
50
100
200
survival
time (day)
C
daGS>TIF-1ARNAi
D
ActGS
-RU486
+RU486
E
daGS
F
UAS-dA43RNAi
G
UAS-TIF-1ARNAi

Figure S2 Reduction in Pol I activity extends lifespan. Related to Figure 1.

(A) Pre-adult lethality resulting from ubiquitous, constitutive expression of *dA43*^{RNAi} or *Tif-1A*^{RNAi}. The frequency of the developmental stage reached is shown as %. Note that flies containing a balancer chromosome were removed from calculations. n= 54 – 107.

(B) Lifespan of females with adult-onset, ubiquitous induction of *dA43*^{RNAi} achieved by feeding a range of RU486 doses: 0 μM (n= 154 dead/4 censored flies), 10 μM (n= 155/1, $p=4x10^{-12}$ to no RU486, *log-rank test*), 50 μM (n= 157/5, $p=4x10^{-8}$), 100 μM (n= 150/1, p>0.05), 200 μM (n= 148/2, $p=3x10^{-5}$).

(C) Lifespan of females with adult-onset, ubiquitous induction of *Tif-1A*^{RNAi} achieved by feeding a range of RU486 doses: 0 μM (n= 115/16), 10 μM (n= 127/14, $p=5x10^{-3}$ to no RU486, *log-rank test*), 50 μM (n= 124/16, $p=2x10^{-3}$), 100 μM (n= 128/15, $p=3x10^{-3}$), 200 μM (n= 131/19, p>0.05).

(D) - (H) Lifespans of control females carrying driver or transgene alone with/without RU486 (n≈ 150, p>0.05, *log-rank test*).

For lifespans, fly genotype is noted in the top right corner of each panel.

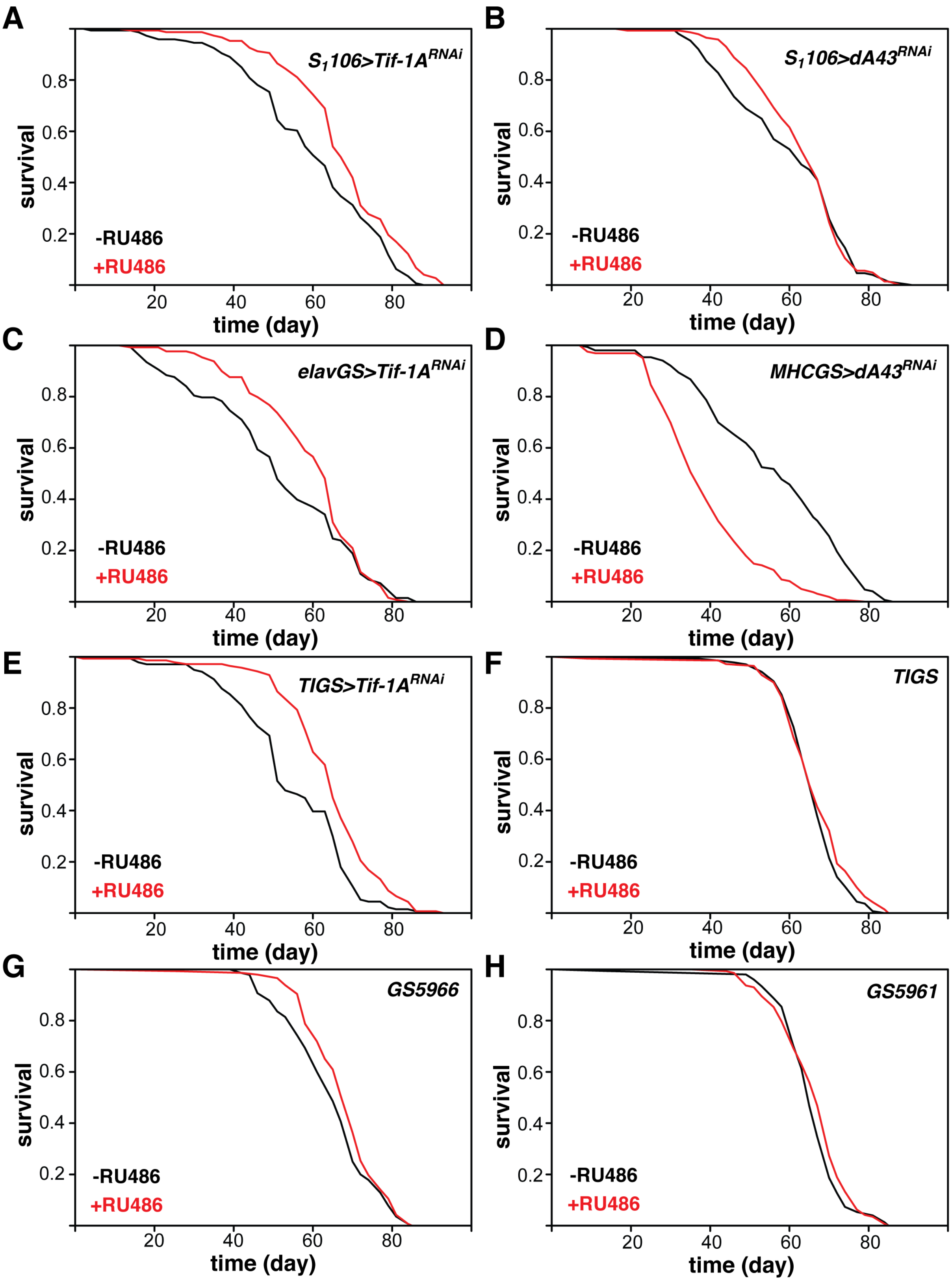

A
$S_1106>Tif\text{-}1A^{RNAi}$
B
$S_1106>dA43^{RNAi}$
C
$elavGS>Tif\text{-}1A^{RNAi}$
D
$MHCGS>dA43^{RNAi}$
E
$TIGS>Tif\text{-}1A^{RNAi}$
F
TIGS
G
GS5966
H
GS5961
survival
0.2
0.4
0.6
0.8
time (day)
20
40
60
80
-RU486
+RU486

Figure S3 Tissue-specific, adult-onset inhibition of Pol I can extend lifespan. Related to Figure 2.

(A) Lifespan of females with adult-onset induction of *dA43*^{RNAi} in fat body and gut achieved by RU486 feeding (-RU486 n= 151 dead/2 censored flies, +RU486 n= 143/1, p>0.05).

(B) Lifespan of females with adult-onset induction of *Tif-1A*^{RNAi} in fat body and gut achieved by RU486 feeding (-RU486 n= 145/2, +RU486 n= 148/2, $p=2x10^{-4}$).

(C) Lifespan of females with adult-onset induction of *Tif-1A*^{RNAi} in neurons achieved by RU486 feeding (-RU486 n= 138/1, +RU486 n= 129/3, p>0.05).

(D) Lifespan of females with adult-onset induction of *dA43*^{RNAi} in muscle achieved by RU486 feeding (-RU486 n= 149/5, +RU486 n= 162/0, $p=5x10^{-21}$).

(E) Lifespan of females with adult-onset, gut-restricted induction of *Tif-1A*^{RNAi} achieved by RU486 feeding (-RU486 n= 135 dead/3 censored, +RU486 n= 139/2, $p=2x10^{-6}$, *log-rank test*).

(F) – (H) Lifespans of control females carrying driver alone with/without RU486 (n≈140, p>0.05, *log-rank test*).

Fly genotype is noted in the top right corner of each panel.

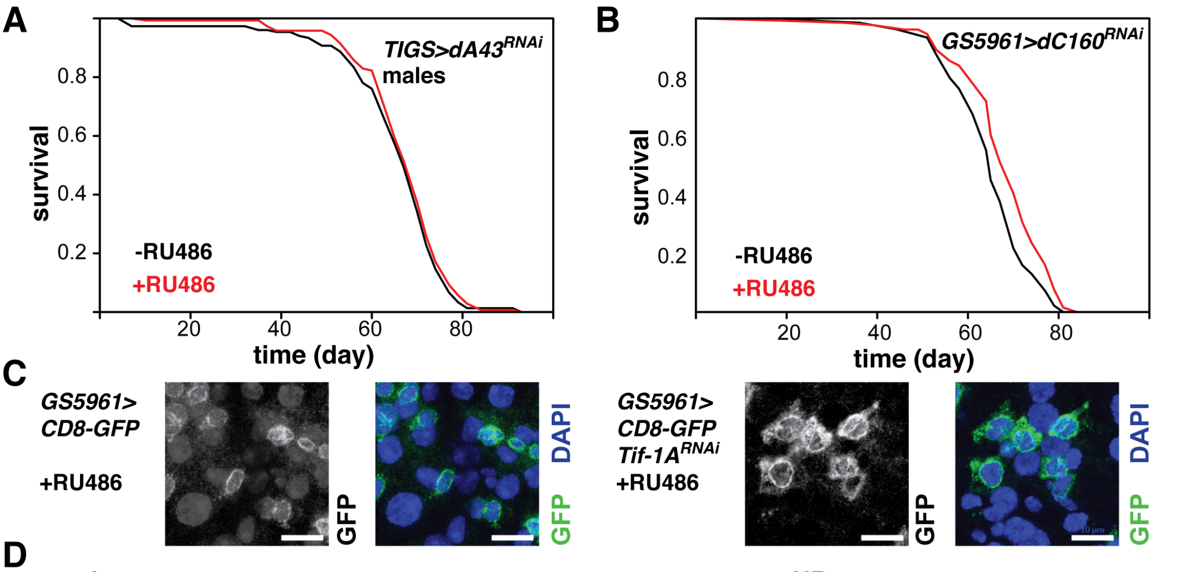

D

covariate	HR	p
**genotype**		
combined Pol I & III *versus* individual Pol I & III	1.30889	4.2×10^{-11}
Pol III *versus* Pol I	0.44783	$<2\times10^{-16}$
**RU486**	0.70339	1.6×10^{-6}
**genotype:RU486**		
combined Pol I and III *versus* individual Pol I & III:RU486	1.00982	0.85
Pol III *versus* Pol I:RU486	0.94065	0.48

E

**Multivariate analysis**		**Individual response variables**			
		moving duration	*velocity*	*time in central zone*	*rotation frequency*
**Covariate**	**p value**				
age	$<10^{-4}$	$<10^{-4}$	$<10^{-4}$	1.6×10^{-3}	$<10^{-4}$
genotype	0.06	0.06	0.77	0.14	0.64
age:genotype	0.01	0.01	0.26	0.93	0.24

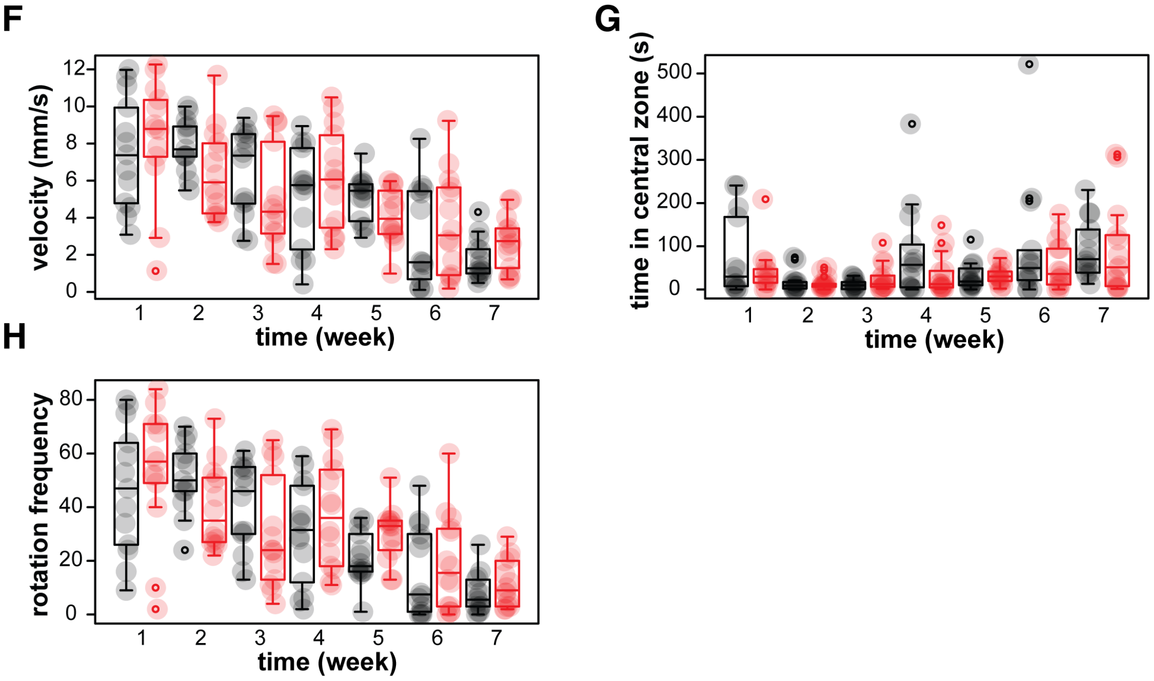

Figure S4 Relationship between Pol I and Pol III in the ISCs and analysis of exploratory walking. Related to Figure 3 and Figure 4.

(A) Induction of *dA43*^{RNAi} in the male gut does not extend lifespan (n≈150, p>0.05, *log-rank test*).

(B) Lifespan of females with adult-onset induction of *dC160*^{RNAi} (targeting Pol III) in the ISCs achieved by RU486 feeding (-RU486 n= 138 dead/3 censored flies, +RU486 n= 131/1, p=1x10^{-3}, *log-rank test*). Note that this lifespan was carried out in parallel with the lifespans presented in Figure 4B. Fly genotype is noted in top right corner in A and B.

(C) *UAS-Tif-1A*^{RNAi} does not interfere with the ability of the *GS5961* driver to induce expression of *UAS-CD8-GFP* in the presence of RU486 (scale bar = 10 μm).

(D) *Cox Proportional Hazards* analysis of the interaction between induction of *dC160*^{RNAi} or *Tif-1A*^{RNAi} in ISCs (total n=770 dead/ 57 censored flies). The model examined the effect of genotype, RU486 and their interaction where the effect of genotype was assessed using two *a priory* contrasts: 1) comparing the genotype containing both RNAi lines to the genotypes containing only one RNAi line, 2) comparing the genotypes containing the single RNAi lines to each other. ":" indicates the interaction term. Note that the lack of significant interaction between genotype and RU486 indicates the lifespan effects of each Pol I are not significantly different from each other or from the combined knockdown. The data included in the model are shown in Figure 3B and Figure S3B.

(E) Full statistical analysis of exploratory walking data. Results of the multivariate LM including the response of individual variables.

(F) – (H) Additional parameters of exploratory walking.
